# Extracorporeal Membrane Oxygenation (ECMO) Cannula Stimulation of the Carotid Sinus Causing Bradycardic Pauses in a Patient With COVID-19

**DOI:** 10.7759/cureus.37652

**Published:** 2023-04-16

**Authors:** John Dayco, Justin Pawloski, Caleb Sokolowski, Dhruvil Patel, Micheal Rits, Grace Goodrich, Saliha Erdem, M. Chadi Alraies

**Affiliations:** 1 Internal Medicine, Wayne State University Detroit Medical Center, Detroit, USA; 2 Internal Medicine, Wayne State University School of Medicine, Detroit, USA; 3 Cardiology, Wayne State University Detroit Medical Center, Detroit, USA

**Keywords:** covid, vv ecmo, electrocardiogram, cardiac assist devices, bradycardia

## Abstract

Veno-venous extracorporeal membrane oxygenation (VV-ECMO) cannulation is a potential cause of episodic bradycardia during an intensive care course because of the proximal cannula insertion site being in the vicinity of the carotid sinus. Herein, we report the case of episodic bradycardia throughout a multi-week intensive care stay of a VV-ECMO recipient due to a severe coronavirus disease 2019 (COVID-19) infection that did not emerge for the rest of the patient's hospitalization after decannulation.

## Introduction

The need for extracorporeal membrane oxygenation (ECMO) in coronavirus disease 2019 (COVID-19) patients has been well-characterized as an important bridge to recovery in individuals with severe respiratory distress [1]. Complications associated with ECMO in the intensive care setting in these patients can range from benign to severe and thus are recommended to be vigilantly observed during their hospital course [2,3]. Herein we discuss the case of an ECMO recipient that continued to have episodic bradycardia during maneuvers and at times, no movement scenarios. Understanding that this can be a potential complication of ECMO can aid clinicians in triaging these concerns appropriately in the context of the patient’s hospital stay. This case was previously presented as a case presentation at the 2023 American College of Cardiology Annual Scientific Meeting on March 5, 2023.

## Case presentation

History of presentation

A 26-year-old female patient with no significant past medical history presented with worsening shortness of breath that began at the start of her day at another hospital. The patient was found to be COVID-19 positive and initially treated with an outpatient course of prednisone. Five days later, the patient returned to that same hospital due to worsening symptoms, with accompanying desaturations requiring a high-flow nasal cannula. The patient continued to be hypoxic, being recorded in the lower to mid 80% oxygen saturation (SpO2). Eventually, a decision to initiate mechanical intubation with vasopressor support was made. Despite the aggressive measures, the patient continued to desaturate, and a decision was made to initiate veno-venous (VV)-ECMO. She was then transferred to our facility to undergo the procedure due to her admitting facility not having ECMO capabilities. She arrived intubated with a blood pressure of 106/75 mm Hg and a heart rate of 104 beats/min. She was afebrile at 36.7°. 21-French and 29-French venous cannulas were inserted into the right internal jugular and right femoral veins, respectively. She tolerated the procedure with no cardiac complications. The patient remained under VV-ECMO support for a total of 33 days. Throughout her VV-ECMO course, the patient developed multiple episodes of sinus bradycardic pauses, lasting for approximately 3-3.5 seconds, beginning on day six since VV-ECMO support began. 

**Figure 1:**
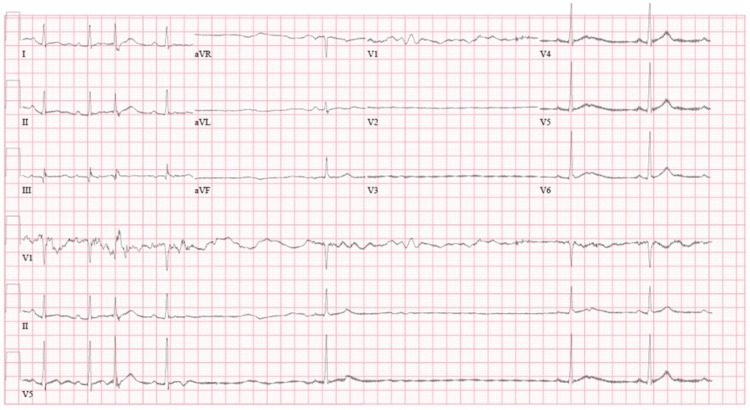
EKG during bradycardic pauses An electrocardiogram (EKG) during the described episode was done, demonstrating a sinus bradycardic pause lasting for 3.5 seconds, after which the patient returned to their baseline rhythm. A similar pattern of pauses was recorded from EKGs taken during subsequent bradycardic pause incidents throughout the patient’s intensive care stay.

**Figure 2:**
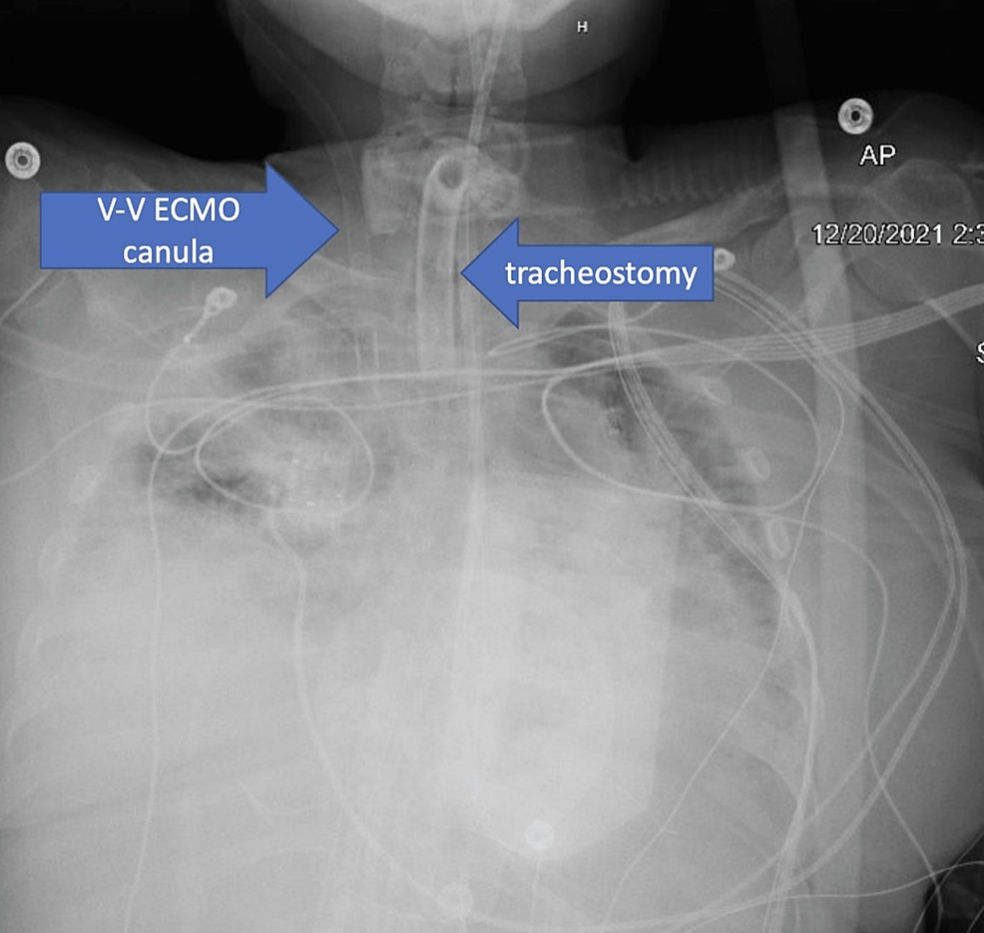
Chest x-ray taken after EKG A chest x-ray was taken during suspicion of possible extracorporeal membrane oxygenation (ECMO) machine issue, following a “chugging” noise near the cannulation site. Read revealed that the veno-venous (VV)-ECMO cannula location in the right, lateral aspect of the cervical neck was correct, and no major adjustments were needed, but the insertion site was noted to be close to the anatomical site of the right carotid sinus.

An electrocardiogram (EKG) obtained during one of these events is shown in Figure 1, which had similar results to EKGs in all other instances throughout her course. Each of these events was accompanied by the VV-ECMO cannula making a “chugging” sound around the lateral right side of the neck, right at the cannula insertion site. A chest x-ray demonstrated the VV-ECMO cannula insertion in the right lateral aspect of the cervical neck (Figure 2) and was thereby determined to be in an adequate position. In addition, the bradycardic pauses would also occur with the mechanical manipulation of the tracheostomy tube, commonly during secretion suctioning. Lastly, the bradycardic pauses would also occur with vagal stimulation, such as the palpation of the VV-ECMO cannula insertion point, or with simple positional manipulation of the patient for decubitus ulcer prevention. After the occurrence of a bradycardic pause, the sinus rhythm would restore to a baseline rate of 55 beats per minute. Due to the benign nature of such pauses and the complications associated with cannula surgical repositioning, no intervention was conducted. The patient had no other incidence of cardiac complications throughout her stay. After 33 days of VV-ECMO support, the patient was eventually decannulated and the bradycardic pauses ceased to recur during the rest of the hospital stay. A carotid sinus massage performed three weeks after decannulation led to a slight bradycardic response, albeit no pauses or presyncope. The patient otherwise did not have any bradycardiac episodes during the rest of the hospitalization stay. 

Differential diagnosis

The differential diagnosis for recurrent bradycardia can be broad. Due to our patient’s admission presentation, and need for intensive care, we narrowed down the likely cause to the following: metabolic source from electrolyte abnormalities including most commonly hyperkalemia, COVID-19 infection-associated bradycardia, induced carotid sinus hypersensitivity from an event occurring during manipulation of cannulation site.

Investigations

We performed an EKG during one of the events, which showed an elongated P-R interval (Figure 1). At this point, the inpatient team also checked for abnormal vitals or electrolyte abnormalities, but no deviations from the patient’s baseline were found. Due to the “chugging” noise heard by the care team, a chest x-ray was also obtained to assess for potential complications from cannulation that may affect device activity and subsequent positioning (Figure 2). The image indicated proper positioning of the VV-ECMO cannula in the right internal jugular vein, and within notable proximity of the anatomical location of the carotid sinus.

Management

Due to the episodic nature of the bradycardic pauses and its benign nature, the care team continued to observe the patient carefully. No medication was necessitated as pauses did not persist to cause any evident clinical deterioration. With the proximity of the internal jugular cannula tip to the sino-atrial node and the insertion site being close to the carotid sinus nervous body, stimulation of these regions by the cannula tubing was the surmised cause for the bradycardic episodes. Minor manipulation of the cannula was attempted to adjust the angle and depth of insertion. This maneuver had limited effect and was not performed routinely due to the risk of stitch collapse and potential unwanted vascular complications. Thereby a conservative approach was concluded by the team, which consisted of observation on telemetry throughout the VV-ECMO course.

Follow-up

Immediately after transfer from the intensive care unit (ICU), the patient recovered in an inpatient rehabilitation unit. The patient tolerated VV-ECMO decannulation and did not require any intensive intervention for the rest of her hospital stay. The patient did not return for any other hospital admission per EMR review since discharge nine months ago.

## Discussion

Many complications are associated with VV-ECMO including bleeding, infection, acute kidney injury, intracerebral hemorrhage, seizure, and stroke. To the best of our knowledge from a review of the literature, the patient we described is the first case of VV-ECMO cannula-induced sinus bradycardia, but similar findings have been reported with other forms of mechanical manipulation around the carotid sinus. While syncope is not a concern for sedated patients, bradyarrhythmia has been shown to increase the risk of thromboembolic complications [4]. Several cases of episodic sinus bradycardia have been observed in laryngoscopic procedures, laryngeal mask airway placements, and invasive squamous cell carcinoma including cases of reproducible bradycardic pauses [5]. In 349 patients who underwent right heart catheterization via the internal jugular vein, two patients experienced episodic sinus bradycardia upon insertion [6]. In a randomized, controlled trial of 240 patients, researchers varied the positive airway pressure while patients had a right internal jugular vein catheter, and the study found a significant increase in hypotension and sinus bradycardia as the pressure increased [7].

In all these cases, the bradycardic episodes were attributed to the mechanical manipulation of the carotid sinus, which lies between the larynx and the internal jugular vein. Sedating medications are believed to increase the risk of reflex bradycardia by lowering the sympathetic tone, and patients can be more susceptible to reflex bradycardia if their sympathetic tone is increased at baseline [8]. In the case of our patient, VV-ECMO cannula placement near the carotid sinus, sedating medications, and positive pressure from the tracheostomy tube likely provoked carotid sinus hypersensitivity. The carotid baroceptors at the bifurcation of the common carotid artery begin the arterial baroreflex with highly sensitive responses to changes in blood pressure to maintain perfusion to the brain. An increase in pressure to the carotid sinus sends an afferent signal to the brainstem, which then sends an efferent signal via the vagus nerve to the SA node to control heart rate [9].

Another possible cause for the patient’s bradycardia is sinus node dysfunction (SND). The most common associations with SND include age, hypertension, diabetes, as well as cardiac defects including right and left bundle branch blocks [10]. COVID-19, however, has emerged as a new association with sinus bradycardia and sinus node dysfunction [11]. While it is plausible that our patient may have been suffering from a previously undiagnosed SND, this cause would not explain the bradycardia and sinus pauses elicited during the movement of the VV-ECMO cannula. 

In addition, there have been reports of central catheter-inducing arrhythmias, as well as cases of insertion leading to sinus arrest or sustained bradycardia [12]. Furthermore, COVID-19 itself has been linked to producing conduction defects, including bradyarrhythmias but the primary mechanism behind this is not well characterized [13]. COVID-19-associated bradycardia is also found to not increase the chance of mortality in a meta-analysis by Umeh et al., which favors prognosis but the relationship between COVID-19 and bradyarrhythmias analyzed in this analysis cannot be ignored and thereby must be considered as a possible cause in diagnostic evaluation [14]. Yet given the patient's clinical course after explanation, with no bradycardic episodes occurring - we determined that the likeliness of primary COVID-induced conduction abnormality is minimal, but worth considering given the growing body of evidence suggesting this relationship. The proposed mechanism in these cases is the mechanical stimulation of the catheter tip into the right atrium in the vicinity of the sinus node, disrupting conduction. In our case, based on x-ray confirmation, the VV-ECMO cannula was near the carotid sinus, while maintaining a reasonable distance from the endocardium. Thereby any relationship with the cardiac conduction system directly was not likely. The only other source remaining was the region near cannulation where carotid sinus anatomy is typically present.

## Conclusions

The case presents an interesting phenomenon of potential VV-ECMO cannula stimulation of the carotid sinus leading to bradycardia and asystole that was suggested after all other pathological causes were ruled out. Although relatively benign in this patient, carotid sinus hypersensitivity due to cannula placement can be a possible etiology of bradycardia in the intensive care unit. Such events may be avoided by optimizing the placement of the VV-ECMO cannula by confirming appropriate landmarks and positioning through imaging. In the absence of other likelier causes, such an event also provides an opportunity for outpatient monitoring and testing to properly evaluate for carotid sinus hypersensitivity, if previously undiagnosed.
